# Rewriting the radiation playbook: NSABP B-51 and the rise of pathologic precision

**DOI:** 10.1038/s41523-025-00819-7

**Published:** 2025-11-05

**Authors:** Mehran Habibi, Bruce G. Haffty, Silvia C. Formenti

**Affiliations:** 1 Division of Breast Surgery, Miami Cancer Institute, Baptist Health Cancer Care, Florida International University, Miami, USA; 2 Department of Radiation Oncology, Rutgers Cancer Institute of New Jersey, New Brunswick, NJ USA; 3 Department of Radiation Oncology, Weill Cornell Medicine, New York, NY USA

**Keywords:** Cancer, Immunology, Oncology

## Abstract

The NSABP B-51/RTOG 1304 trial represents a paradigm shift in breast cancer management, showing that patients achieving nodal clearance after neoadjuvant chemotherapy derive no added benefit from regional nodal irradiation. These results challenge long-standing practice, promotes de-escalation, and raise biologically significant questions, particularly regarding immunologic mechanisms and clinical subgroup variations.

## Introduction and contextualization

In the evolving era of biologically guided oncology, the NSABP B-51/RTOG 1304 trial, recently published by Mamounas and colleagues in the *New England Journal of Medicine*^1^, stands as a landmark moment: not only for its findings, but for its philosophy. By demonstrating that regional nodal irradiation (RNI) offers no apparent oncologic benefit for patients with initially node-positive breast cancer who convert to pathologically node-negative (ypN0) status after neoadjuvant chemotherapy, the authors challenge decades of radiation practice and reaffirm the primacy of response-guided treatment. It also offers some hypothesis-generating insight on the mechanisms behind the findings.

## Clinical efficacy and toxicity

The authors should be commended for the precision of the trial’s design and the discipline of its execution. Radiation planning was based on CT simulation and subjected to centralized quality assurance. Adverse event monitoring was comprehensive, and the absence of unexpected toxicity enhances the credibility of the findings. Grade 3 dermatitis occurred in 10.3% of patients in the RNI group versus 6.5% in the no-RNI group, a clinically meaningful difference. But perhaps their greatest contribution is philosophical: a recalibration of how we think about risk, benefit, and the architecture of treatment.

This study convincingly answers a practical clinical question that has long hovered in the space between guideline, judgment, and bias rooted in institutional practice patterns, training, or interpretation of retrospective literature: does a patient who presents with N1 disease, sterilized by systemic therapy, still require nodal irradiation? The answer, based on this well-executed trial, is no; though some important questions remain.

The trial met its rigorous design objectives and had high protocol adherence (97% completed their assigned radiation regimen), with a five-year event rate of invasive breast cancer recurrence or death of 2.9% in the no-RNI group versus 2.1% in the RNI group (hazard ratio, 0.88; *P* = 0.51). Locoregional recurrence was uncommon in both groups (1.4% vs. 0.8%), and there were no significant differences in distant recurrence, disease-free survival, or overall survival. While the follow-up is limited to five years, relatively short for hormone receptor–positive disease, the absolute number of events was low in both arms. This limits the likelihood that large absolute differences would emerge in the ensuing years. Furthermore, as CDK4/6 inhibitors become standard in high-risk HR+ disease, the baseline recurrence risk may decline further, potentially diminishing the marginal benefit of regional radiation in this population^2,3^. Though a non-significant trend toward benefit in HR+ patients was observed (HR 0.72; 95% CI 0.49–1.06), this requires continued observation.

This is a practice-altering study. And it lands at a moment when breast oncology is increasingly defined not by what we add, but by what we safely omit. Just as the ACOSOG Z0011^4^ and AMAROS^5^ trials shifted the axillary surgery paradigm for patients with minimal nodal disease, and as MINDACT^6^ and TAILORx^7^ have enabled endocrine therapy to replace chemotherapy for many genomically low-risk patients, NSABP B-51 invites us to abandon an outdated heuristic: that initial clinical stage should dictate final local-regional treatment.

## Nodal vs. breast pathologic response

Notably, about 20% of patients in the trial had residual invasive disease in the breast while achieving a pathologic complete response in the axilla. Even in this subgroup, traditionally considered high-risk due to residual tumor burden and often receiving additional boost, the omission of regional nodal irradiation did not compromise outcomes^1^. This finding challenges the long-standing assumption that residual breast disease mandates regional radiation and instead elevates nodal response as the more clinically consequential endpoint. The implication is clear: in the modern era of response-guided care, it is the eradication of nodal disease—not breast pCR—that best predicts regional control and survival.

## Implications for high-risk subsets

This shifts the treatment compass decisively from “where disease began” to “how disease responded.” Even in patients with T3 N1 tumors, 14.3% of the B-51 cohort, who underwent mastectomy and had no residual nodal disease, the study protocol permitted no radiation at all in the control arm, no chest wall radiation, no nodal irradiation; and yet these patients fared as well as their radiated counterparts. The trial also included a meaningful proportion of patients under age 50, reinforcing the relevance of these findings even in groups historically considered at higher risk. In the setting of robust pathologic clearance, additional radiation is unnecessary and may be unwarranted.

One might expect resistance to such a conclusion, particularly from radiation oncology, where postoperative chest wall and regional nodal radiation have been foundational. While current guidelines still recommend radiation in patients with T3 tumors or initial N1 disease, the trial results presented here are likely to modify those guidelines.

## Historical perspective and guidelines

Historically, the rationale for RNI in patients with nodal involvement derived from large meta-analyses demonstrating a disease-free survival benefit^8^. These studies attributed the effect to improvements in local control. Yet as early as 2008, it was proposed that this survival advantage might also be immunologically mediated—through a mechanism now understood as immunogenic cell death^9^. Radiation’s potential to prime dendritic cell–mediated antitumor immunity suggests that its role extends beyond cytotoxicity, offering systemic effects that enhance tumor visibility to the immune system.

## Immunologic considerations and future hypotheses

Interestingly, in the B-51 trial in patients with TNBC under the age of 49—a subgroup often considered to have the most aggressive disease—RNI was associated with a higher hazard ratio for recurrence (HR 2.30), suggesting a possible detrimental effect^1^. Indeed, emerging preclinical data now indicate that irradiation of tumor-draining lymph nodes (TDLNs) can impair systemic immune responses, particularly those dependent on antigen presentation by conventional type 1 dendritic cells (cDC1s)^10–13^. These findings are intriguing in view of the recent evidence on the importance of preserving intact tumor drainage, sustaining immune fitness to achieve the best outcomes in the setting of immunotherapy^14,15^.

Although the B-51 cohort largely preceded the era of routine immunotherapy, the trial’s demonstration that nodal pCR alone obviates the need for RNI—even in the presence of residual breast disease—raises compelling questions. Might radiation to tumor-draining lymph nodes, in future cohorts receiving immunotherapy, impede the immune system’s capacity to clear minimal residual disease? As immunotherapy increasingly enters early-stage settings, such interactions between radiation fields and immune function warrant careful exploration.

## Caveats and limitations

These findings should not be generalized to patients with residual nodal disease, more than three positive nodes at diagnosis, inflammatory breast cancer, or T4 tumors, who were not included in the B-51 trial.

## Conclusion

In summary, this study marks a turning point for the common subset of patients with up to 3 nodes involved at diagnosis. It does not suggest that radiation is obsolete—only that its use should be tailored based on the evolving biology of the treated cancer. As with the Z0011, TAILORx, and MINDACT trials, NSABP B-51 invites us to reimagine not only the content of our treatments, but the principles by which we justify them. While there remain some unanswered questions—such as a potential benefit in women with ER/PR + /HER2- tumors, or whether women with residual IHC+ disease derive any benefit—it is important to recognize that the risk of local-regional relapse was exceedingly low in the arm that received no regional nodal radiation (11/784 or 1.4%), indicating this group of patients does exceedingly well without regional nodal or PMRT.

It is incumbent on clinicians—surgeons, medical oncologists, and radiation oncologists alike—to ensure that practice evolves with evidence. We owe that to our patients, whose risk of recurrence is now more accurately measured not by where their disease began, but by how it evolves with treatment.

## Data Availability

No datasets were generated or analysed during the current study.
